# Racialized economic segregation and inequities in treatment initiation and survival among patients with metastatic breast cancer

**DOI:** 10.1007/s10549-024-07319-5

**Published:** 2024-05-03

**Authors:** Harlan Pittell, Gregory S. Calip, Amy Pierre, Cleo A. Ryals, Jenny S. Guadamuz

**Affiliations:** 1 Flatiron Health, 233 Spring St, New York, NY 10013 USA; 2 Program on Medicines and Public Health, University of Southern California School of Pharmacy, Los Angeles, USA; 3 Division of Hematologic Malignancies, Department of Medicine, Memorial Sloan Kettering Cancer Center, New York, NY USA; 4 Present Address: Division of Health Policy and Management, Berkeley School of Public Health, University of California, Berkeley, USA

**Keywords:** Racialized economic segregation, Index of concentration at the extremes, Structural racism, Breast cancer, Metastatic breast cancer

## Abstract

**Purpose:**

Racialized economic segregation, a form of structural racism, may drive persistent inequities among patients with breast cancer. We examined whether a composite area-level index of racialized economic segregation was associated with real-world treatment and survival in metastatic breast cancer (mBC).

**Methods:**

We conducted a retrospective cohort study among adult women with mBC using a US nationwide electronic health record-derived de-identified database (2011–2022). Population-weighted quintiles of the index of concentration at the extremes were estimated using census tract data. To identify inequities in time to treatment initiation (TTI) and overall survival (OS), we employed Kaplan–Meier methods and estimated hazard ratios (HR) adjusted for clinical factors.

**Results:**

The cohort included 27,459 patients. Compared with patients from the most privileged areas, those from the least privileged areas were disproportionately Black (36.9% vs. 2.6%) or Latinx (13.2% vs. 2.6%) and increasingly diagnosed with de novo mBC (33.6% vs. 28.9%). Those from the least privileged areas had longer median TTI than those from the most privileged areas (38 vs 31 days) and shorter median OS (29.7 vs 39.2 months). Multivariable-adjusted HR indicated less timely treatment initiation (HR 0.87, 95% CI 0.83, 0.91, *p* < 0.01) and worse OS (HR 1.19, 95% CI 1.13, 1.25, *p* < 0.01) among those from the least privileged areas compared to the most privileged areas.

**Conclusion:**

Racialized economic segregation is a social determinant of health associated with treatment and survival inequities in mBC. Public investments directly addressing racialized economic segregation and other forms of structural racism are needed to reduce inequities in cancer care and outcomes.

**Supplementary Information:**

The online version contains supplementary material available at 10.1007/s10549-024-07319-5.

## Introduction

Structural racism is defined as the “differential access to the goods, services, and opportunities of society by race” perpetuating the widespread and unfair treatment of people of color through institutions, practices, and laws [1, 2]. Its impact can be felt in a number of domains, including access to housing, education, and healthcare [3]. A notable manifestation of structural racism is racialized economic segregation—a persistent byproduct of the unfair institutional practice of redlining—which has been associated with inequities in health and healthcare access, including among patients with cancer [4–6].

Racialized economic segregation is hypothesized to impact the incidence of cancer and cancer outcomes through its related inequities in access to healthcare facilities, adverse environmental exposures, and the built environment [7]—an association consistently demonstrated across multiple health outcomes with known inequities that exist between Black and White patients [8]. This study offers an in-depth analysis of the implications of racialized economic segregation for patients with metastatic breast cancer (mBC)—a cancer with well-documented racial and ethnic inequities in incidence, treatment, and survival [9, 10].

Building upon related studies that have highlighted the impact of socioeconomic status (SES)—especially lower SES—along with other social determinants of health (SDOH) on health inequities [11–13], our research aims to deepen the understanding of how racialized economic segregation contributes to these health inequities. Previous research has shown that measures of residential segregation, redlining and structural racism relate to key outcomes for those with breast cancer, including stage at diagnosis, treatment with surgery, and mortality (both all cause and cancer specific) [4, 5, 14–18]. Collectively, such studies have demonstrated that women with breast cancer who reside in economically marginalized neighborhoods have often experienced notably poorer outcomes, particularly, worse survival. Here, we build on earlier research in several notable ways. First, we examined data from a national cohort, which expands upon previous studies that have examined patient cohorts from individual states, including Maryland, Florida, and New Jersey. Second, we analyzed a contemporary patient cohort that includes those diagnosed with mBC after the onset of the Covid-19 pandemic. Such evidence is needed given other evidence of worsening racial and ethnic inequities during this time period [19]. Third, unique to this study is its examination of treatment initiation, a previously unexplored outcome in the context of racialized economic segregation. This investigation is critical, as timely initiation of treatment is a key factor influencing prognosis and survival [20]. Finally, our study makes a methodological contribution by comparing the sensitivity of our results to the choice and construction of measures of our key exposure. As related studies have examined several different measures of structural racism and racialized economic segregation, it is unclear whether their results are influenced by the specific measure chosen, potentially impacting the comparability of results across studies. Through these contributions, our study addresses several key gaps in the existing literature and underscores the need for policies and interventions that are sensitive to these socioeconomic dimensions, thereby enhancing healthcare outcomes for patients with mBC.

## Methods

### Data source

**Fig. 1:**
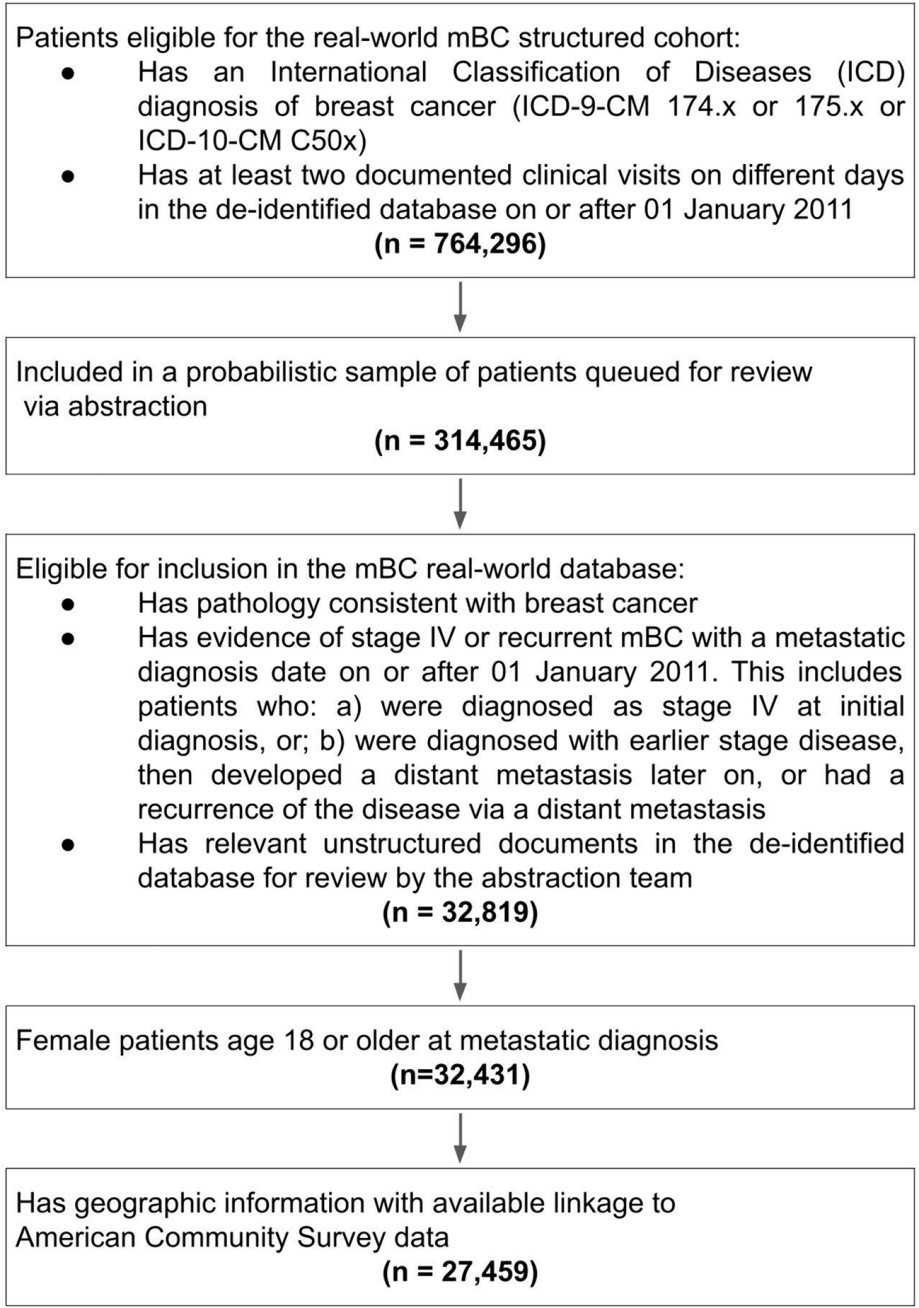
Cohort selection diagram. Patients are probabilistically sampled to ensure that an adequate number of patients are queued for chart review during the abstraction phase. A computer-based algorithm for probabilistic selection is used. A small number of patients had an electronic health record of the event of interest prior to the index date and such patients were excluded from those analyses. This is typically a data entry error (e.g., a practice may schedule a treatment that the patient is not able to receive or the date of death is incorrect). *mBC* metastatic breast cancer

This study used the nationwide Flatiron Health electronic health record (EHR)-derived, de-identified database—a longitudinal database comprising de-identified patient-level structured and unstructured data, curated via technology-enabled abstraction [21, 22]. During the study period, the de-identified data originated from approximately 280 US cancer clinics (approximately 800 sites of care). The study included 27,459 adult women diagnosed with mBC from January 01, 2011 to December 31, 2022 and included women with early-stage breast cancer that later metastasized and de novo mBC [23]. See Fig. 1 for a detailed cohort selection diagram.

### Variables and endpoints

Using data from the American Community Survey (2015–2019, 5-year estimates), we constructed four neighborhood-level measures of racialized economic segregation and SES. Following an approach used by related studies, we categorized each measure into US population-weighted quintiles, with Quintile 1 denoting the least privileged census tracts and Quintile 5 denoting the most privileged census tracts [24, 25]. Our main results focused on the index of concentration at the extremes (ICE), a geospatial measure of racialized economic segregation distinguishing between the least and most privileged groups in an area [23]. By focusing on ICE, the exposure of interest in related research on the implications of racialized economic segregation for health, including mBC outcomes, we aim to contextualize our results within this broader body of literature [4, 5, 17, 18]. Consistent with the terminology used in the original work describing the ICE methodology, we employ the terms “least privileged” versus “most privileged” to distinguish between social groups that have experienced systematic oppression versus systematic advantage from racialized economic segregation.

Thus, our measure of ICE corresponded to the concentration of low-income Black households (least privileged group) and high-income White households (most privileged group) within a census tract following prior studies. Low-income Black households consisted of non-Latinx Black households with incomes below $25,000 per year, and high-income White households consisted of non-Latinx White households with incomes of $100,000 or greater. These income thresholds correspond to the bottom and top quintiles for US household income during the specified years [26]. As a sensitivity analysis, we examined two additional constructions of ICE that compared the concentration of high-income White households to low-income Latinx households and low-income households of Color. Our secondary analysis examined three additional area-level SDOH measures related to racialized economic segregation: (1) The percent of a census tract reporting as non-Latinx Black (Percent Black); (2) The Yost Index, a measure of SES derived from seven inputs reflecting educational attainment, income, housing, and employment within an area [27]; and (3) The Structural Racism Indicator—a newer, composite index of racialized economic segregation based on education, income, household structure, employment, public assistance, occupation, and racial and ethnic composition [28].

Patients included in the cohort were followed from metastatic diagnosis to the first event of interest, death, or last confirmed activity. Time to treatment initiation (TTI) was defined as the start of first-line systemic therapy as evidenced by an EHR-documented order or administration of an antineoplastic therapy determined by oncologist-defined, rule-based lines of therapy. TTI has been recognized as a “patient-centered quality metric” that reflects timely care and has been linked to health outcomes in mBC, including survival [10, 29]. Overall survival (OS) was defined based on documented mortality status. Mortality information was curated from available EHR sources, including structured and unstructured data (e.g., clinician notes). Additionally, the EHR data are linked with commercial obituary data and data from the US Social Security Death Index [30] to supplement dates of death not documented in the EHR. This measure of OS reflects all-cause mortality, as the data sources utilized do not allow for differentiation between cancer-specific and other causes of death.

At the patient level, race and ethnicity values were categorized into mutually exclusive groups: Latinx, non-Latinx Asian (hereafter, Asian), non-Latinx Black (hereafter, Black), non-Latinx White (hereafter White), and Other/Not documented. This latter group included patients without an EHR-documented race or ethnicity and patients with a value of “Other Race.” In the study database, the “Other Race” value is the result of Flatiron Health’s data de-identification process, which masked specific race categories with lower representation in the U.S. population, including American Indian or Alaska Native, Native Hawaiian or Other Pacific Islander, and patients with multiple races.

### Statistical analyses

The Kaplan–Meier product-limit estimator was used to compare TTI and OS between patients residing in areas with differing levels of racialized economic segregation (as measured using ICE quintiles). The Cox model was used to estimate hazard ratios (HR), adjusting for clinical factors, of TTI and OS with a reference group fixed to those residing in the most privileged neighborhoods. HR below one indicated a lower likelihood of initiating treatment (indicative of less timely treatment) and a lower likelihood of death (indicative of increased survival), while HR above one indicated a higher likelihood of initiating treatment and a higher likelihood of death.

The choice of clinical characteristics for adjustment—age at metastatic diagnosis (continuous), year of metastatic diagnosis (categorical), stage at initial diagnosis, molecular subtype, number of metastases, sites of metastasis, and Eastern Cooperative Group (ECOG) performance status—was informed by, and is an extension of, a conceptual framework for health care inequities published in 2003 by the Institute of Medicine (now the National Academy of Medicine) [31]. This framework recognizes that, on average, People of Color have lower socioeconomic profiles than Whites and that such differences in socioeconomic status contribute to inequities in both healthcare access and health outcomes. Thus, according to this framework, socioeconomic status mediates racial/ethnic inequities and, therefore, should not be included as an adjuster in models quantifying the extent of racial/ethnic inequities. Accordingly, we did not adjust for measures of socioeconomic status, like health insurance, as adjustment for socioeconomic status would mitigate the estimated “independent effect” of racialized economic segregation on our outcomes of interest [32, 33].

We also estimated both interactive and stratified models by race and ethnicity using White patients residing in the most privileged areas as the reference group. As an exploratory analysis, we repeated our analysis examining three alternate measures of racialized economic segregation described above. To better characterize the cohort, we also summarized patients’ characteristics by race and ethnicity and examined the distribution of first-line treatments among patients initiating treatment. Statistical analyses were conducted using RStudio Version 2022.12.0 + 353 with R Version 4.2.2 [34, 35]. We tested the proportionality assumption of our Cox proportional hazards models by inspecting Schoenfeld residual plots, employing the cox.zph function within the survival package in R [36].

## Results

### Characteristics of the overall cohort

Table 1 summarizes the characteristics of the 27,459 patients in the cohort. Median age at metastatic diagnosis was 64 (IQR 54–73). Grouped by stage, 57.3% of patients were diagnosed with early-stage breast cancer that later metastasized, 31.0% presented with de novo breast cancer, and 11.8% had an unknown stage. Among breast cancer subtypes, hormone receptor-positive (HR+)/human epidermal growth factor receptor 2-negative (HER2−) was the most prevalent, representing 66.1% of cases. This was followed by HR+/HER2+ at 12.3%, HR−/HER2− at 11.6%, and HR−/HER2+ at 4.0%. A small portion, 6.1% of patients, had an unknown subtype. By practice type, 79.9% of patients were associated with community oncology practices, while 20.1% were associated with academic practices. Supplemental Table S1 presents a comparison of the characteristics by race and ethnicity. Compared to White patients, Latinx and Black patients were younger (median age: Latinx = 58, Black = 60, White = 65) more likely to reside in the least privileged areas (ICE Q1: Latinx = 36.2%, Black = 58.4%, White = 7.6%) and differed in insurance coverage (% with Medicaid coverage: Latinx = 5.8%, Black = 4.4%, White = 1.5%). Information on the distribution of first-line treatment among the cohort can be found in Supplemental Table S2. The most common first-line treatment was Aromatase inhibitors (32.1%), followed by chemotherapy (19.6%), Aromatase inhibitors + cyclin-dependent kinase 4/6 inhibitors (17.7%) with the remainder of patients receiving other therapies (30.6%).

**Table 1:** Demographic and clinical characteristics of the mBC cohort at diagnosis: overall and by neighborhood privilege

	All patients *N* = 27,459	Q1: least privileged *N* = 4734	Q2 *N* = 5045	Q3 *N* = 5405	Q4 *N* = 5706	Q5: most privileged *N* = 6569
Age [IQR]	64.0 [54.0–73.0]	62.0 [52.0–72.0]	64.0 [54.0–73.0]	64.0 [54.0–73.0]	64.0 [54.0–74.0]	64.0 [54.0–74.0]
Age group, *n* (%)						
19–34	599 (2.2%)	147 (3.1%)	125 (2.5%)	98 (1.8%)	112 (2.0%)	117 (1.8%)
35–49	4014 (14.6%)	761 (16.1%)	753 (14.9%)	761 (14.1%)	819 (14.4%)	920 (14.0%)
50–64	9781 (35.6%)	1815 (38.3%)	1781 (35.3%)	1968 (36.4%)	1959 (34.3%)	2258 (34.4%)
65–74	7017 (25.6%)	1119 (23.6%)	1322 (26.2%)	1352 (25.0%)	1515 (26.6%)	1709 (26.0%)
≥ 75	6048 (22.0%)	892 (18.8%)	1064 (21.1%)	1226 (22.7%)	1301 (22.8%)	1565 (23.8%)
Race and ethnicity, *n* (%)						
Latinx	1722 (6.3%)	623 (13.2%)	476 (9.4%)	250 (4.6%)	199 (3.5%)	174 (2.6%)
Asian	548 (2.0%)	76 (1.6%)	120 (2.4%)	114 (2.1%)	102 (1.8%)	136 (2.1%)
Black	2994 (10.9%)	1747 (36.9%)	492 (9.8%)	314 (5.8%)	270 (4.7%)	171 (2.6%)
White	15,566 (56.7%)	1177 (24.9%)	2614 (51.8%)	3333 (61.7%)	3795 (66.5%)	4647 (70.7%)
Other/not documented	6629 (24.1%)	1111 (23.5%)	1343 (26.6%)	1394 (25.8%)	1340 (23.5%)	1441 (21.9%)
Practice type, *n* (%)						
Academic	5510 (20.1%)	778 (16.4%)	772 (15.3%)	821 (15.2%)	1138 (19.9%)	2001 (30.5%)
Community oncology	21,949 (79.9%)	3956 (83.6%)	4273 (84.7%)	4584 (84.8%)	4568 (80.1%)	4568 (69.5%)
Insurance type, *n* (%)						
Commercial	5969 (21.7%)	973 (20.6%)	990 (19.6%)	1170 (21.6%)	1285 (22.5%)	1551 (23.6%)
Medicaid	658 (2.4%)	224 (4.7%)	157 (3.1%)	132 (2.4%)	85 (1.5%)	60 (0.9%)
Medicare	13,785 (50.2%)	2205 (46.6%)	2554 (50.6%)	2723 (50.4%)	2929 (51.3%)	3374 (51.4%)
Other/Unknown	7047 (25.7%)	1332 (28.1%)	1344 (26.6%)	1380 (25.5%)	1407 (24.7%)	1584 (24.1%)
ECOG performance status, *n* (%)						
0	10,402 (37.9%)	1690 (35.7%)	1834 (36.4%)	2071 (38.3%)	2213 (38.8%)	2594 (39.5%)
1	7281 (26.5%)	1295 (27.4%)	1410 (27.9%)	1486 (27.5%)	1538 (27.0%)	1552 (23.6%)
2+	3259 (11.9%)	612 (12.9%)	633 (12.5%)	666 (12.3%)	675 (11.8%)	673 (10.2%)
Not documented	6517 (23.7%)	1137 (24.0%)	1168 (23.2%)	1182 (21.9%)	1280 (22.4%)	1750 (26.6%)
Diagnosis year, *n* (%)						
2011–2013	5780 (21.0%)	973 (20.6%)	1067 (21.1%)	1099 (20.3%)	1245 (21.8%)	1396 (21.3%)
2014–2016	7460 (27.2%)	1193 (25.2%)	1409 (27.9%)	1502 (27.8%)	1535 (26.9%)	1821 (27.7%)
2017–2019	7871 (28.7%)	1395 (29.5%)	1429 (28.3%)	1540 (28.5%)	1630 (28.6%)	1877 (28.6%)
2020–2022	6348 (23.1%)	1173 (24.8%)	1140 (22.6%)	1264 (23.4%)	1296 (22.7%)	1475 (22.5%)
Group stage, *n* (%)						
Early stage	15,721 (57.3%)	2566 (54.2%)	2840 (56.3%)	3111 (57.6%)	3336 (58.5%)	3868 (58.9%)
De novo	8505 (31.0%)	1592 (33.6%)	1617 (32.1%)	1680 (31.1%)	1717 (30.1%)	1899 (28.9%)
Not documented	3233 (11.8%)	576 (12.2%)	588 (11.7%)	614 (11.4%)	653 (11.4%)	802 (12.2%)
Number of metastases, *n* (%)						
1	8893 (32.4%)	1550 (32.7%)	1661 (32.9%)	1717 (31.8%)	1902 (33.3%)	2063 (31.4%)
2	7000 (25.5%)	1215 (25.7%)	1292 (25.6%)	1375 (25.4%)	1422 (24.9%)	1696 (25.8%)
3+	11,447 (41.7%)	1946 (41.1%)	2070 (41.0%)	2295 (42.5%)	2359 (41.3%)	2777 (42.3%)
Not documented	119 (0.4%)	23 (0.5%)	22 (0.4%)	18 (0.3%)	23 (0.4%)	33 (0.5%)
Site of metastasis, *n* (%)						
Bone only	4947 (18.0%)	786 (16.6%)	912 (18.1%)	980 (18.1%)	1110 (19.5%)	1159 (17.6%)
Visceral	7671 (27.9%)	1484 (31.3%)	1439 (28.5%)	1449 (26.8%)	1532 (26.8%)	1767 (26.9%)
Other	122 (0.4%)	30 (0.6%)	16 (0.3%)	13 (0.2%)	34 (0.6%)	29 (0.4%)
Not documented	14,719 (53.6%)	2434 (51.4%)	2678 (53.1%)	2963 (54.8%)	3030 (53.1%)	3614 (55.0%)
Molecular subtype, *n* (%)						
HR+/HER2−	18,138 (66.1%)	2873 (60.7%)	3274 (64.9%)	3572 (66.1%)	3911 (68.5%)	4508 (68.6%)
HR+/HER2+	3385 (12.3%)	604 (12.8%)	626 (12.4%)	687 (12.7%)	668 (11.7%)	800 (12.2%)
HR−/HER2−	3183 (11.6%)	738 (15.6%)	617 (12.2%)	603 (11.2%)	577 (10.1%)	648 (9.9%)
HR−/HER2+	1087 (4.0%)	228 (4.8%)	208 (4.1%)	216 (4.0%)	219 (3.8%)	216 (3.3%)
Not tested/unknown	1666 (6.1%)	291 (6.1%)	320 (6.3%)	327 (6.0%)	331 (5.8%)	397 (6.0%)

*ECOG* Eastern Cooperative Oncology Group, *HR* hormone receptor status, *HER2* Human Epidermal Growth Factor Receptor 2 status, *IQR* interquartile range, *mBC* metastatic breast cancer

Race and ethnicity values are mutually exclusive groups with Asian, Black, White, and Other/not documented denoting non-Latinx patients. Other/not documented includes patients without an EHR-documented race and ethnicity and patients with a recorded value of “Other Race.” Due to small cohort sizes consistent with current representation in the US population, American Indian or Alaska Native and Native Hawaiian or Pacific Islander race values were grouped into the “Other Race” category. Health insurance status was defined as Medicare for patients age 65 or older at metastatic diagnosis. For the remainder of patients, health insurance status denoted their EHR-documented insurance record closest to their metastatic diagnosis date. For patients with multiple records on the same data, the following hierarchy was used: Medicare, Commercial, Other, and Medicaid. Number of metastases denotes the distinct sites of metastasis identified through abstraction, covering 18 sites, including bone, lung, liver, brain, thyroid, and spleen, among others. Molecular subtype denotes closest value within ± 90 days of metastatic diagnosis

### Characteristics of the cohort by neighborhood privilege

Table 1 summarizes the characteristics of the cohort by neighborhood privilege. The smallest share of the cohort came from the least privileged neighborhoods (17.2%), and the largest share came from the most privileged neighborhoods (24.0%). Compared to patients from the most privileged areas, those from the least privileged areas exhibited several differences. They were typically younger (median age: 62 vs 64), more likely to be Black (36.9% vs 2.6%) or Latinx individuals (13.2% vs 2.6%) were less often diagnosed with early-stage disease (54.2% vs 58.9%) and more frequently had the aggressive HR−/HER2− subtype (15.6% vs 9.9%).

### Median time and adjusted HR of treatment initiation

**Fig. 2:**
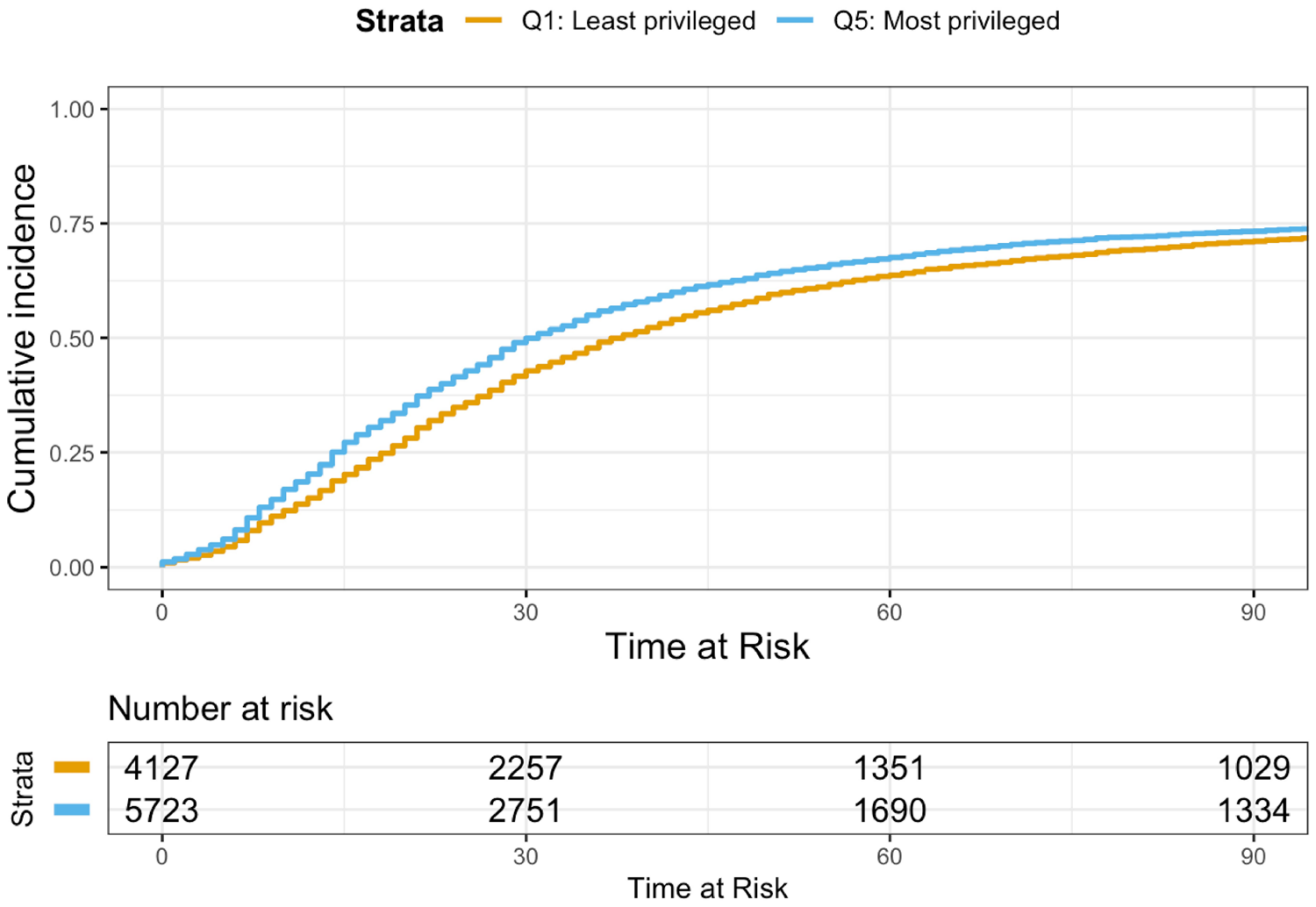
Kaplan–Meier estimates of treatment initiation comparing patients from the least and most privileged neighborhoods. Treatment initiation denotes first-line treatment initiation within 90 days of metastatic diagnosis. Plot generated in R using the survminer package (version 0.4.9). For ease of interpretation, this figure was limited to two curves—comparing the least and most privileged areas—and plotted the inverse risk of initiating first-line treatment (to denote that more patients received treatment as time progressed). Treatment initiation estimates excluded 3463 patients with a recorded therapy starting prior to metastatic disease that continued beyond 14 days after the index date of metastatic disease

Figure 2 depicts Kaplan–Meier curves of the risk of initiating first-line treatment within 90 days of metastatic diagnosis by neighborhood privilege. For ease of interpretation, we limited the figure to two curves—comparing the least and most privileged areas—and plotted the inverse risk of initiating first-line treatment (to denote that more patients received treatment as time progressed). At nearly all points in time, patients in the least privileged areas (represented by the orange line) had a lower risk of initiating treatment than patients in the most privileged areas (represented by the blue line).

Table 2 presents median TTI by neighborhood privilege. Patients in the least privileged areas had a longer median TTI than patients in the most privileged areas (38 days, 95% CI 36, 40 vs 31 days, 95% CI 29, 32). In addition, those in the least privileged areas had an adjusted HR indicative of less timely treatment initiation (HR 0.905, 95% CI 0.863, 0.950) relative to those in the most privileged areas (reference group).

**Table 2:** Kaplan–Meier estimates and hazard ratios of treatment initiation and overall survival by neighborhood privilege

ICE quintile	Median time to first-line treatment initiation in days	Unadjusted hazard ratio of risk of first-line treatment initiation	Adjusted hazard ratio of risk of first-line treatment initiation
*I. Treatment initiation [CI]*			
Q1: least privileged	38 [36, 40]	0.917*** [0.879, 0.958]	0.905*** [0.863, 0.950]
Q2	36 [35, 38]	0.920*** [0.882, 0.960]	0.890*** [0.852, 0.929]
Q3	34 [32, 35]	0.948** [0.91, 0.988]	0.897*** [0.861, 0.936]
Q4	32 [31, 34]	0.966* [0.928, 1.006]	0.928*** [0.891, 0.967]
Q5: most privileged	31 [29, 32]	Reference	Reference
*II. Overall survival [CI]*			
Q1: least privileged	29.7 [28.5, 31.5]	1.250*** [1.190, 1.313]	1.170*** [1.107, 1.237]
Q2	33.4 [31.8, 34.9]	1.138*** [1.084, 1.194]	1.124*** [1.070, 1.182]
Q3	34.0 [32.8, 35.4]	1.143*** [1.090, 1.198]	1.126*** [1.073, 1.181]
Q4	36.0 [34.7, 37.4]	1.066*** [1.017, 1.116]	1.076*** [1.027, 1.128]
Q5: most privileged	39.2 [37.9, 40.6]	Reference	Reference

*ICE* index of concentration at the extremes

Median time to event was estimated using Kaplan–Meier methods, and hazard ratios were estimated using the Cox model. 95% confidence intervals (CI) are reported in brackets. Adjusted hazard ratios were adjusted for age at metastatic diagnosis (continuous), year of metastatic diagnosis (categorical), stage at initial diagnosis, molecular subtype, number of metastases, sites of metastasis, and Eastern Cooperative Oncology Group performance status. The proportionality assumption for the Cox proportional hazards models was evaluated through visual inspection of Schoenfeld residuals plots, provided by the cox.zph function from the survival package. Plots for each variable remained close to horizontal, indicating that the assumption was reasonably met. Treatment initiation estimates excluded 3463 patients with a recorded therapy starting prior to metastatic disease that continued beyond 14 days after the index date of metastatic disease. Overall survival estimates excluded 181 patients whose recorded death occurred before the index date of metastatic diagnosis

***p < 0.01; **p < 0.05, *p < 0.10

### Median time and adjusted HR of OS

**Fig. 3:**
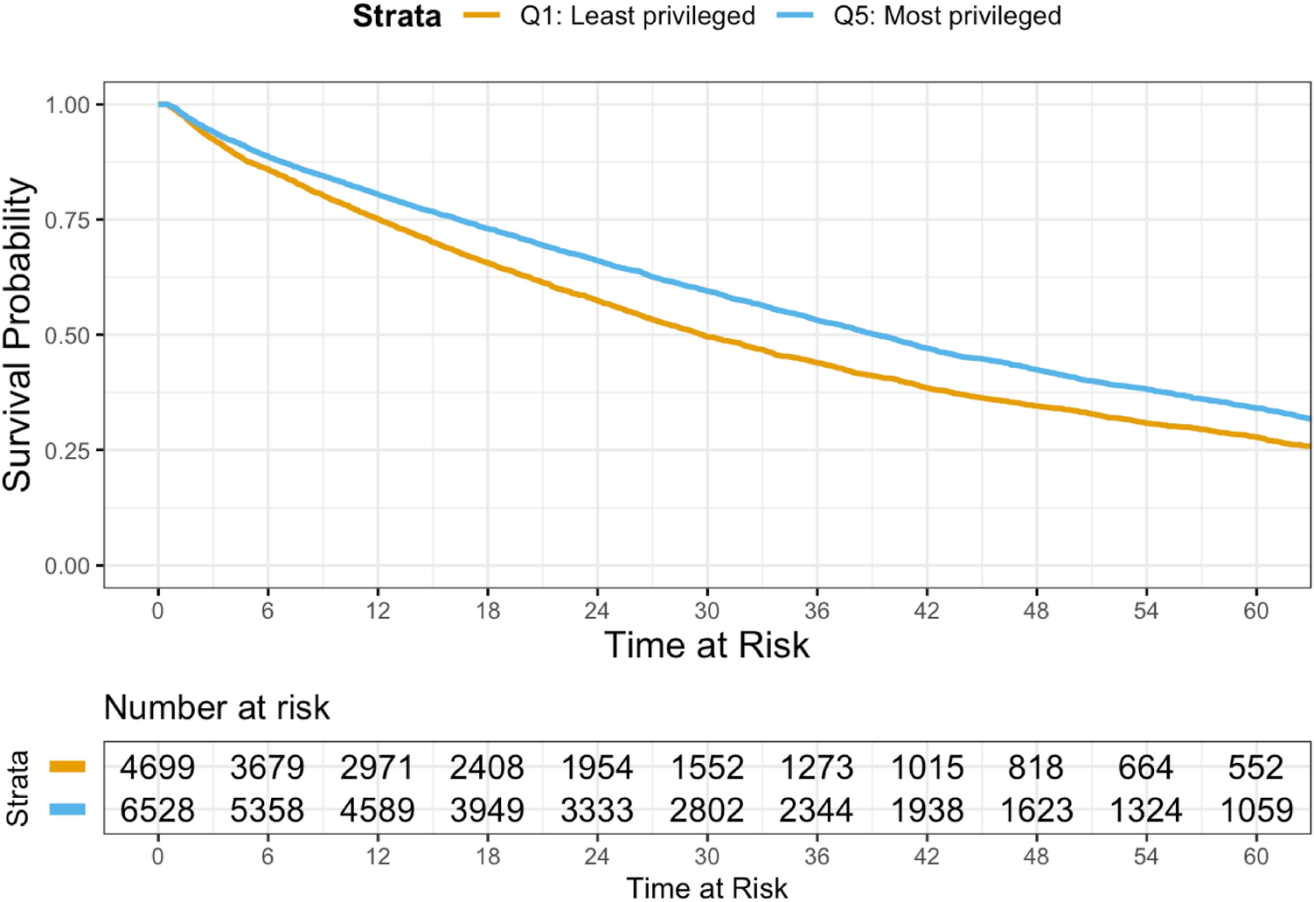
Kaplan–Meier survival estimates comparing patients from the least and most privileged neighborhoods. Kaplan–Meier survival estimates within 5 years of metastatic diagnosis. Survival estimates excluded 181 patients whose recorded death occurred before the index date of metastatic diagnosis. Such exclusion likely results from recording the date of death as year-month for privacy reasons and the use of middle of the month in time-to-event calculations. Plot generated in R using the survminer package (version 0.4.9)

Figure 3 depicts Kaplan–Meier curves of the risk of death within 5 years of metastatic diagnosis by neighborhood privilege. At nearly all points in time, patients in the least privileged areas (represented by the orange line) had a greater risk of death than patients in the most privileged areas (represented by the blue line).

Table 2 presents median OS from metastatic diagnosis by neighborhood privilege. Patients in the least privileged areas had shorter median OS time (29.7 months, 95% CI 28.5, 31.5) than patients in the most privileged areas (39.2 months, 95% CI 37.9, 40.6). In addition, those in the least privileged areas had an adjusted HR indicative of an increased risk of death (HR 1.170, 95% CI 1.107, 1.237) relative to those in the most privileged areas (reference group).

### Interactive and stratified results by patient race and ethnicity

Table 3 presents the results from adjusted hazard models incorporating an interaction term between neighborhood privilege and race and ethnicity. Within these models, the reference group denoted White patients from the most privileged neighborhoods. Relative to this group, nearly all other groups had HR indicative of a lower risk of initiating first-line treatment and a greater risk of death. In our analysis of TTI, HR were consistently below one for Black and Asian patients and were similar among those in more and less privileged areas. For Latinx and White patients, HR of TTI were in all but one instance below one, but were often closer to one among those in more privileged areas. In our analysis of OS, HR for Black and White patients were closer to one among those residing in more privileged neighborhoods. Among Asian and Latinx patients, trends across survival HR were less consistent.

**Table 3:** Adjusted hazard ratios with interactions between ICE quintile, race, and ethnicity

ICE quintile	Latinx	Asian	Black	White	Other/unknown
*I. Adjusted hazard ratios of first-line treatment initiation [CI]*					
Q1: least privileged	0.755*** [0.685, 0.833]	0.855 [0.653, 1.119]	0.822*** [0.770, 0.877]	0.876*** [0.814, 0.943]	0.792*** [0.733, 0.857]
Q2	0.756*** [0.676, 0.844]	0.942 [0.768, 1.155]	0.875** [0.786, 0.974]	0.872*** [0.825, 0.922]	0.722*** [0.672, 0.776]
Q3	0.779*** [0.674, 0.899]	0.846 [0.688, 1.040]	0.843** [0.735, 0.967]	0.884*** [0.839, 0.930]	0.739*** [0.688, 0.793]
Q4	0.911 [0.775, 1.071]	0.693*** [0.554, 0.868]	0.794*** [0.686, 0.918]	0.910*** [0.867, 0.956]	0.792*** [0.738, 0.850]
Q5: most privileged	1.017 [0.855, 1.210]	0.845* [0.694, 1.028]	0.826** [0.693, 0.984]	Reference	0.794*** [0.741, 0.851]
*II. Adjusted hazard ratios of death [CI]*					
Q1: least privileged	0.945 [0.839, 1.066]	0.801 [0.565, 1.135]	1.276*** [1.188, 1.371]	1.160*** [1.068, 1.260]	1.359*** [1.242, 1.487]
Q2	1.060 [0.928, 1.209]	1.059 [0.824, 1.361]	1.268*** [1.126, 1.428]	1.108*** [1.040, 1.180]	1.152*** [1.061, 1.251]
Q3	0.954 [0.798, 1.142]	0.764* [0.576, 1.014]	1.249*** [1.082, 1.443]	1.127*** [1.064, 1.194]	1.178*** [1.086, 1.278]
Q4	1.056 [0.864, 1.291]	0.663** [0.483, 0.910]	1.420*** [1.216, 1.658]	1.089*** [1.030, 1.151]	1.036 [0.954, 1.125]
Q5: most privileged	0.983 [0.791, 1.222]	0.788* [0.606, 1.024]	1.136 [0.939, 1.374]	Reference	1.018 [0.939, 1.104]

*ICE* index of concentration at the extremes

Adjusted hazard ratios estimated using the Cox model with adjustment for age at metastatic diagnosis (continuous), year of metastatic diagnosis (categorical), ECOG performance status, group stage at initial diagnosis, molecular subtype, number of metastasis sites, and site of metastasis. 95% confidence interval (CI) in brackets. Treatment initiation estimates excluded 3463 patients with a recorded therapy starting prior to metastatic disease that continued beyond 14 days after the index date of metastatic disease. Overall survival estimates excluded 181 patients whose recorded death occurred before the index date of metastatic diagnosis

***p < 0.01; **p < 0.05; *p < 0.10

Supplementary Table S3 presents Kaplan–Meier estimates of treatment initiation stratified by neighborhood privilege and race and ethnicity and offers additional evidence of racial and ethnic inequities in TTI and rwOS. For example, median TTI was 29 days (95% CI 28, 31) among White patients from the most privileged areas compared to 47 days (95% CI 42, 56) among Latinx patients from the least privileged areas and 38 days (95% CI 35, 40) among Black patients from the least privileged areas. Likewise, for Asian, Black, and White patients, median rwOS was lowest among those from the least privileged areas. Supplementary Table S4 provides evidence of similar inequities in results from adjusted HR of TTI and rwOS stratified by race and ethnicity.

### Associations with additional social determinants of health measures

Supplementary Table S5 presents adjusted HR of treatment initiation and OS using alternate constructions of ICE. Compared to our main results, which defined ICE in relation to high-income White and low-income Black households in an area, our results using ICE constructed in relation to low-income Latinx households and low-income households of Color offered similar evidence of inequities. For example, across these three constructions, HR among those from the least privileged areas ranged from 0.872 (95% CI 0.830, 0.915) to 0.905 (95% CI 0.863, 0.950) for TTI and from 1.143 (95% CI 1.081, 1.209) to 1.170 (95% CI 1.107, 1.237) for rwOS.

Table 4 lists correlations between ICE and three additional SDOH measures related to structural racism and SES. These additional measures exhibited strong, positive correlations (> 0.65) with one another, except for Percent Black and the Yost Index (correlation of 0.380). This weaker correlation may reflect the absence of an economic component in the Percent Black measure and the absence of a racial component in the Yost Index.

**Table 4:** Correlations among additional social determinants of health measures

	Index of concentration at the extremes	Percent Black	Yost Index	Structural racism indicator
Index of concentration at the extremes	1	–	–	–
Percent Black	0.699	1	–	–
Yost Index	0.837	0.380	1	–
Structural Racism Indicator	0.868	0.679	0.735	1

Correlations denote Pearson correlation coefficients

Table 5 presents the results from an exploratory analysis that utilized these additional measures of structural racism and SES. The top panel presents adjusted HR of the risk of first-line treatment initiation. Among those from neighborhoods with varying concentrations of Black residents, with the exception of Q3, we found no difference in the risk of treatment initiation as all confidence intervals included one. Using the Yost Index, our results were similar to those from our main analysis of ICE, as patients from areas with the lowest SES (Yost Index Quintile 1) had a lower risk of treatment initiation (HR 0.902, 95% CI 0.861, 0.946) than those from the highest SES (Yost Index Quintile 5). Analyses leveraging the Structural Racism Indicator also revealed evidence of inequities in treatment initiation with a lower risk of treatment initiation among those from areas with the highest levels of structural racism (HR 0.954, 95% CI 0.909, 1.002), though the confidence interval included one.

**Table 5:** Adjusted hazard ratios of treatment initiation and overall survival using alternate measures of structural racism and socioeconomic status

Quintile	Index of concentration at the extremes	Percent Black	Yost Index	Structural Racism Indicator
*I. Adjusted hazard ratios of risk of first-line treatment initiation [CI]*				
Q1: least privileged	0.905*** [0.863, 0.950]	1.017 [0.967, 1.069]	0.902*** [0.861, 0.946]	0.954* [0.909, 1.002]
Q2	0.890*** [0.852, 0.929]	1.012 [0.967, 1.059]	0.898*** [0.859, 0.938]	0.922*** [0.881, 0.964]
Q3	0.897*** [0.861, 0.936]	1.068*** [1.022, 1.117]	0.919*** [0.882, 0.959]	0.938*** [0.898, 0.979]
Q4	0.928*** [0.891, 0.967]	1.017 [0.973, 1.064]	0.953** [0.915, 0.992]	0.971 [0.932, 1.012]
Q5: most privileged	Reference	Reference	Reference	Reference
*II. Adjusted hazard ratios of risk of death [CI]*				
Q1: least privileged	1.170*** [1.107, 1.237]	1.071** [1.012, 1.134]	1.173*** [1.112, 1.237]	1.180*** [1.117, 1.247]
Q2	1.124*** [1.070, 1.182]	1.023 [0.971, 1.078]	1.147*** [1.091, 1.206]	1.118*** [1.063, 1.177]
Q3	1.126*** [1.073, 1.181]	1.031 [0.980, 1.084]	1.122*** [1.070, 1.177]	1.112*** [1.059, 1.167]
Q4	1.076*** [1.027, 1.128]	0.992 [0.943, 1.043]	1.059** [1.011, 1.110]	1.053** [1.005, 1.104]
Q5: most privileged	Reference	Reference	Reference	Reference

*SDOH* social determinants of health

Adjusted hazard ratios were estimated using the Cox model. 95% confidence intervals (CI) are reported in brackets. Adjusted hazard ratios were adjusted for age at metastatic diagnosis (continuous), year of metastatic diagnosis (categorical), stage at initial diagnosis, molecular subtype, number of metastases, sites of metastasis, and Eastern Cooperative Oncology Group performance status. Treatment initiation estimates excluded 3463 patients with a recorded therapy starting prior to metastatic disease that continued beyond 14 days after the index date of metastatic disease. Overall survival estimates excluded 181 patients whose recorded death occurred before the index date of metastatic diagnosis

***p < 0.01; **p < 0.05; *p < 0.10

The bottom panel of Table 5 presents adjusted HR of the risk of death. Similar to our main analysis of ICE, our analysis of additional, area-level measures revealed evidence of inequities in OS when comparing the top and bottom quintiles of each measure. Across models, HR among those from areas in the bottom quintile were 1.071 (95% CI 1.012, 1.134) using the Percent Black measure, 1.173 (95% CI 1.112, 1.237) using the Yost Index, and 1.180 (95% CI 1.117, 1.247) using the Structural Racism Indicator.

## Discussion

This study offers robust evidence of the association between racialized economic segregation and treatment initiation and survival inequities among patients with mBC. Consistent with prior research [4, 5, 15], patients from less privileged areas were disproportionately Black or Latinx and increasingly diagnosed with de novo mBC. Our analysis, employing a neighborhood-level measure of segregation, showed that patients from less privileged areas faced delayed treatment initiation and shorter survival compared to patients from more privileged neighborhoods, as indicated by both Kaplan–Meier estimates and adjusted HR. Such results are consistent with earlier studies of breast cancer cohorts which examined individual patient data from three states [5, 17, 18] and found that residence in less privileged areas as defined using ICE was associated with an increased risk of death with similar results from an analysis of a national cohort of county-level data [4]. While differences in treatment initiation were more difficult to discern among Asian and Latinx patients due to wider confidence intervals (i.e., smaller sample sizes), sharp inequities were evident among Black patients, especially those from the least privileged areas. Notably, Black patients from even the most privileged areas experienced shorter median survival times compared to White patients living in the least privileged neighborhoods, underscoring the deep impact of racialized economic segregation on OS among patients with mBC. Further evidence of these findings comes from our exploratory analyses using alternate SDOH measures related to structural racism, which showed similar inequities. These results collectively underscore the critical influence that racialized economic segregation plays in shaping health outcomes for patients with mBC.

The historical consequences of racialized economic segregation have been long-lasting and profound through its contribution to limited upward mobility and the wealth gap between different racial and ethnic groups [6, 37]. Our study substantially expands the understanding of its consequences on health from past studies in several ways. First, we examined how racialized economic segregation related to both treatment initiation and survival, building on past studies which solely focused on investigating inequities in survival [4, 5]. Second, we utilized a tract-level measure of racialized economic segregation, differing from a prior study that examined a less granular county-level measure of segregation [4]. An advantage of tract-level data is its potential to offer a more nuanced reflection of racialized economic segregation, given that census tracts are small, relatively permanent statistical subdivisions of a county with a lower median population size accounting for within-county segregation [38]. Third, we examined multiple alternative measures of segregation in our sensitivity analysis, providing a significant methodological contribution. Findings from that analysis suggest that while multiple approaches can be used to measure the implications of racialized economic segregation among patients with mBC, the choice of these approaches should be aligned with a conceptual framework that takes into account the relevant associations of interest. Finally, we examined data from a national and contemporary cohort of patients from across the USA, building on some of the past studies that relied on data from a single state during the pre-pandemic period [17].

There are several ways in which structural racism influences cancer care and cancer outcomes in the USA, and understanding such pathways is essential for designing effective solutions [11–13, 39–41]. Environmental injustices like the disproportionate placement of power plants near communities of color amplify cancer risks for those communities [42]. The structure of the US healthcare system acts as a barrier to preventive cancer screenings, with Latinx and Black adults being three times less likely to have insurance compared to White adults [43]. Policies that divert healthcare resources away from predominantly Black neighborhoods, like closures of publicly funded hospitals, also contribute to this inequity [44]. With respect to our findings, racialized economic segregation may influence health preferences and behaviors, inequities in the healthcare marketplace within communities, and discrepancies in environmental risk factors affecting health needs. A considerable proportion of the observed racial and ethnic inequities in breast cancer are attributed to geographic-level factors that are linked to the racial and ethnic composition of neighborhoods and communities [45]. Additionally, racial and ethnic inequities in the stage at which breast cancer is diagnosed likely reflects differential access to early screening and preventive measures, highlighting the urgent need for policies promoting equitable healthcare access. Ultimately, reducing racial and ethnic inequities in cancer care and cancer outcomes requires public investments in areas that directly address structural racism (e.g., enhancing funding for medical facilities in segregated neighborhoods, implementing policies for equitable healthcare reimbursements across different communities, and establishing community health outreach programs to improve access).

### Limitations

This study has its limitations. As with any observational study, there is potential for unmeasured confounders to influence our survival estimates as notably, our dataset lacked comprehensive information on comorbidities. Additionally, our analysis was limited to a measure of all-cause mortality, precluding a more precise examination of cancer-related mortality, although a related study examining both all-cause and cancer-specific mortality found similar results across outcomes [15]. The residential location, census tract, for patients was geocoded to the most recent address documented in the EHR, and measures of racialized economic segregation may not fully capture this experience at diagnosis, throughout a patient’s cancer journey, or before diagnosis. Our analysis was also focused on female patients with mBC, given the limited number of male patients in our dataset, which introduces uncertainty about the generalizability of our results to all patients with mBC, including male patients and those diagnosed with early-stage disease. Finally, the generalizability of our results to other cancers and diseases also remains uncertain. While our results suggest that multiple measures of neighborhood structural racism can be used to detect inequities among patients with mBC, further research is needed to establish the interchangeability of these measures in research on other disease states. Nevertheless, concerns about the generalizability of our results across the USA are mitigated by a prior analysis comparing the study dataset to two other national cohorts, SEER and NPCR, which found these mBC cohorts had similar clinical and demographic characteristics [21].

## Conclusion

Utilizing the index of concentration at the extremes, a measure of racialized economic segregation, we found that patients residing in the least privileged areas experienced a longer median time before initiating first-line therapy for mBC and a shorter median survival than patients in the most privileged areas. These findings were consistent after adjusting for demographic and clinical factors. Notably, we observed that Black patients in less privileged areas were more likely to experience worse outcomes compared to White patients in the most privileged ones. Our study underscores the importance of adopting policies that specifically address structural racism, as they hold the potential to enhance outcomes for patients with mBC.

### Supplementary Information

Below is the link to the electronic supplementary material.

Supplementary file1 (DOCX 38 KB)

## Data Availability

The data that support the findings of this study were originated by and are the property of Flatiron Health, Inc., which has restrictions prohibiting the authors from making the data set publicly available. Requests for data sharing by license or by permission for the specific purpose of replicating results in this manuscript can be submitted to publicationdataaccess@flatiron.com.
