# Supplementary material for: Racialized economic segregation and inequities in treatment initiation and survival among patients with metastatic breast cancer
Source: Breast Cancer Res Treat. 2024 May 3;206(2):411–23. doi: 10.1007/s10549-024-07319-5 (PMC11182814; doi:10.1007/s10549-024-07319-5)
Supplement: Supplementary file 1 — Supplementary file1 (DOCX 38 KB) [file 10549_2024_7319_MOESM1_ESM.docx]

**Racialized economic segregation and inequities in treatment initiation and survival among patients with metastatic breast cancer**

Harlan Pittell, Gregory S. Calip, Amy Pierre, Cleo A. Ryals, Jenny S. Guadamuz

**Corresponding author:** Harlan Pittell, PhD, Flatiron Health, 233 Spring St, New York, NY 10013; email: [harlan.pittell@flatiron.com](mailto:harlan.pittell@flatiron.com)

*Breast Cancer Research and Treatment*

# Supplementary Information

## **Supplementary Table S1** Patient characteristics by race and ethnicity

|  | **Latinx** | **Asian** | **Black** | **White** | **Other/Not documented** |
| --- | --- | --- | --- | --- | --- |
|  | *N=1722* | *N=548* | *N=2994* | *N=15566* | *N=6629* |
| ICE Quintile: |  |  |  |  |  |
| Q1: Least privileged | 623 (36.2%) | 76 (13.9%) | 1747 (58.4%) | 1177 (7.6%) | 1111 (16.8%) |
| Q2 | 476 (27.6%) | 120 (21.9%) | 492 (16.4%) | 2614 (16.8%) | 1343 (20.3%) |
| Q3 | 250 (14.5%) | 114 (20.8%) | 314 (10.5%) | 3333 (21.4%) | 1394 (21.0%) |
| Q4 | 199 (11.6%) | 102 (18.6%) | 270 (9.0%) | 3795 (24.4%) | 1340 (20.2%) |
| Q5: Most privileged | 174 (10.1%) | 136 (24.8%) | 171 (5.7%) | 4647 (29.9%) | 1441 (21.7%) |
| Age | 58.0 [48.0;69.0] | 58.0 [49.0;67.2] | 60.0 [51.0;70.0] | 65.0 [55.0;74.0] | 64.0 [54.0;74.0] |
| Age Group: |  |  |  |  |  |
| 19-34 | 64 (3.7%) | 18 (3.3%) | 112 (3.7%) | 279 (1.8%) | 126 (1.9%) |
| 35-49 | 430 (25.0%) | 125 (22.8%) | 531 (17.7%) | 1997 (12.8%) | 931 (14.0%) |
| 50-64 | 655 (38.0%) | 229 (41.8%) | 1199 (40.0%) | 5420 (34.8%) | 2278 (34.4%) |
| 65-74 | 313 (18.2%) | 110 (20.1%) | 679 (22.7%) | 4221 (27.1%) | 1694 (25.6%) |
| 75+ | 260 (15.1%) | 66 (12.0%) | 473 (15.8%) | 3649 (23.4%) | 1600 (24.1%) |
| Practice Type: |  |  |  |  |  |
| Academic | 382 (22.2%) | 119 (21.7%) | 733 (24.5%) | 3875 (24.9%) | 401 (6.0%) |
| Community oncology | 1340 (77.8%) | 429 (78.3%) | 2261 (75.5%) | 11691 (75.1%) | 6228 (94.0%) |
| Insurance type: |  |  |  |  |  |
| Commercial | 375 (21.8%) | 165 (30.1%) | 748 (25.0%) | 3435 (22.1%) | 1246 (18.8%) |
| Medicaid | 100 (5.8%) | 15 (2.7%) | 131 (4.4%) | 234 (1.5%) | 178 (2.7%) |
| Medicare | 639 (37.1%) | 189 (34.5%) | 1287 (43.0%) | 8216 (52.8%) | 3454 (52.1%) |
| Other/Unknown | 608 (35.3%) | 179 (32.7%) | 828 (27.7%) | 3681 (23.6%) | 1751 (26.4%) |
| ECOG Performance Status: |  |  |  |  |  |
| 0 | 614 (35.7%) | 245 (44.7%) | 1183 (39.5%) | 6080 (39.1%) | 2280 (34.4%) |
| 1 | 450 (26.1%) | 139 (25.4%) | 811 (27.1%) | 4167 (26.8%) | 1714 (25.9%) |
| 2+ | 160 (9.3%) | 58 (10.6%) | 410 (13.7%) | 1780 (11.4%) | 851 (12.8%) |
| Not documented | 498 (28.9%) | 106 (19.3%) | 590 (19.7%) | 3539 (22.7%) | 1784 (26.9%) |
| Year of Metastatic Diagnosis: |  |  |  |  |  |
| 2011-2013 | 346 (20.1%) | 102 (18.6%) | 547 (18.3%) | 3415 (21.9%) | 1370 (20.7%) |
| 2014-2016 | 445 (25.8%) | 159 (29.0%) | 797 (26.6%) | 4518 (29.0%) | 1541 (23.2%) |
| 2017-2019 | 505 (29.3%) | 160 (29.2%) | 909 (30.4%) | 4361 (28.0%) | 1936 (29.2%) |
| 2020-2022 | 426 (24.7%) | 127 (23.2%) | 741 (24.7%) | 3272 (21.0%) | 1782 (26.9%) |
| Region: |  |  |  |  |  |
| Midwest | 62 (3.6%) | 45 (8.2%) | 207 (6.9%) | 2286 (14.7%) | 620 (9.4%) |
| Northeast | 222 (12.9%) | 84 (15.3%) | 301 (10.1%) | 2614 (16.8%) | 969 (14.6%) |
| South | 531 (30.8%) | 107 (19.5%) | 1655 (55.3%) | 4991 (32.1%) | 2736 (41.3%) |
| West | 523 (30.4%) | 193 (35.2%) | 98 (3.3%) | 1772 (11.4%) | 1872 (28.2%) |
| Not documented | 384 (22.3%) | 119 (21.7%) | 733 (24.5%) | 3903 (25.1%) | 432 (6.5%) |
| Group Stage: |  |  |  |  |  |
| De novo | 520 (30.2%) | 167 (30.5%) | 938 (31.3%) | 4613 (29.6%) | 2267 (34.2%) |
| Early stage | 974 (56.6%) | 304 (55.5%) | 1717 (57.3%) | 9272 (59.6%) | 3454 (52.1%) |
| Not documented | 228 (13.2%) | 77 (14.1%) | 339 (11.3%) | 1681 (10.8%) | 908 (13.7%) |
| Number of Metastases: |  |  |  |  |  |
| 1 | 548 (31.8%) | 156 (28.5%) | 895 (29.9%) | 5024 (32.3%) | 2270 (34.2%) |
| 2 | 423 (24.6%) | 141 (25.7%) | 734 (24.5%) | 3963 (25.5%) | 1739 (26.2%) |
| 3+ | 750 (43.6%) | 249 (45.4%) | 1349 (45.1%) | 6516 (41.9%) | 2583 (39.0%) |
| Not documented | 1 (0.1%) | 2 (0.4%) | 16 (0.5%) | 63 (0.4%) | 37 (0.6%) |
| Site of Metastasis: |  |  |  |  |  |
| Bone only | 264 (15.3%) | 73 (13.3%) | 418 (14.0%) | 2905 (18.7%) | 1287 (19.4%) |
| Visceral | 573 (33.3%) | 171 (31.2%) | 990 (33.1%) | 4053 (26.0%) | 1884 (28.4%) |
| Other | 5 (0.3%) | 1 (0.2%) | 10 (0.3%) | 78 (0.5%) | 28 (0.4%) |
| Not documented | 880 (51.1%) | 303 (55.3%) | 1576 (52.6%) | 8530 (54.8%) | 3430 (51.7%) |
| Molecular Subtype: |  |  |  |  |  |
| Luminal A | 1052 (61.1%) | 334 (60.9%) | 1759 (58.8%) | 10728 (68.9%) | 4265 (64.3%) |
| Luminal B | 245 (14.2%) | 83 (15.1%) | 364 (12.2%) | 1868 (12.0%) | 825 (12.4%) |
| TNBC | 230 (13.4%) | 71 (13.0%) | 576 (19.2%) | 1489 (9.6%) | 817 (12.3%) |
| HER2-overexpressing | 97 (5.6%) | 26 (4.7%) | 140 (4.7%) | 550 (3.5%) | 274 (4.1%) |
| Not tested / Unknown | 98 (5.7%) | 34 (6.2%) | 155 (5.2%) | 931 (6.0%) | 448 (6.8%) |

Race and ethnicity values are mutually exclusive groups with Asian, Black, White, and Other/Not documented denoting non-Latinx patients. Other/Not documented includes patients without a documented race and ethnicity and patients with a recorded value of “Other Race.” Due to small cohort sizes consistent with current representation in the US population, American Indian or Alaska Native and Native Hawaiian or Pacific Islander race values were grouped into the "Other Race" category. Health insurance status was defined as Medicare for patients age 65 or older at metastatic diagnosis. For the remainder of patients, health insurance status denoted their EHR-documented insurance record closest to their metastatic diagnosis date. For patients with multiple records on the same data, the following hierarchy was used: Medicare, Commercial, Other, Medicaid. Number of metastases denotes the distinct sites of metastasis identified through abstraction, covering 18 sites including bone, lung, liver, brain, thyroid, and spleen, among others. Molecular subtype denotes closest value within +/- 90-days of metastatic diagnosis. ECOG=Eastern Cooperative Oncology Group. HR=Hormone Receptor status. HER2=Human Epidermal Growth Factor Receptor 2 status. IQR=Interquartile range. mBC=Metastatic breast cancer.

## **Supplementary Table S2** Distribution of patients across first-line therapy classes

| **Therapy Class** | **Share of patients receiving specified first-line therapy** |
| --- | --- |
| Aromatase inhibitor (AI) | 32.1% |
| Chemotherapy | 19.6% |
| AI + Cyclin-dependent kinase (CDK) 4/6 Inhibitors | 17.7% |
| Selective Estrogen Receptor Degrader (SERD) + CDK 4/6 Inhibitors | 7.1% |
| SERD | 6.9% |
| Selective Estrogen Receptor Modulators (SERM) | 6.1% |
| Chemotherapy + HER2-Targeted Therapy | 4.9% |
| Clinical Study Drug | 2.9% |
| CDK 4/6 Inhibitor | 1.4% |
| HER2-Targeted Therapy | 1.3% |

The table summarizes the distribution of patients by first-line therapy class among the 20 most common first-line treatments over the study period. Clinical study lines included at least one clinical study drug.

## **Supplementary Table S3** Kaplan-Meier estimates of treatment initiation and death by neighborhood privilege and race and ethnicity

| **I. Median time to first-line treatment initiation in days** [CI] | | | | | |
| --- | --- | --- | --- | --- | --- |
| ICE Quintile | Asian | Black | Latinx | White | Other / Not documented |
| Q1: Least privileged | 32 [25, 49] | 38 [35, 40] | 47 [42, 56] | 33 [30, 36] | 39 [35, 43] |
| Q2 | 33 [26, 46] | 37 [34, 42] | 43 [37, 51] | 34 [32, 36] | 40 [36, 43] |
| Q3 | 33 [27, 43] | 36 [30, 46] | 49 [37, 59] | 32 [30, 34] | 35 [33, 38] |
| Q4 | 42 [25, 103] | 40 [32, 50] | 33 [28, 41] | 32 [30, 34] | 32 [29, 35] |
| Q5: Most privileged | 44 [28, 71] | 34 [28, 45] | 30 [24, 43] | 29 [28, 31] | 35 [32, 38] |
| **II. Median time to death in months** [CI] | | | | | |
| ICE Quintile | Asian | Black | Latinx | White | Other / Not documented |
| Q1: Least privileged | 40.5 [30.5, NR] | 26.6 [24.8, 28.6] | 43.6 [38.0, 52.9] | 32.0 [29.1, 35.2] | 26.8 [24.0, 30.5] |
| Q2 | 44.6 [35.3, 60.7] | 27.2 [23.7, 31.7] | 40.5 [36.5, 47.4] | 34.1 [32.0, 36.2] | 31.1 [28.8, 34.4] |
| Q3 | 54.7 [39.5, 73.3] | 26.7 [22.2, 34.6] | 42.2 [35.3, 49.4] | 34.8 [33.4, 36.4] | 30.3 [27.8, 33.5] |
| Q4 | 72.7 [38.8, NR] | 27.9 [21.5, 35.5] | 42.3 [33.4, 52.2] | 35.9 [34.3, 37.8] | 36.5 [33.9, 38.9] |
| Q5: Most privileged | 51.8 [41.4, 70.6] | 30.5 [23.8, 39.8] | 36.4 [31.7, 47.5] | 40.0 [38.3, 41.3] | 37.4 [33.4, 40.7] |

Median times to events were estimated using Kaplan-Meier analysis. 95% confidence intervals (CI) are reported in brackets. Bounds not reached denoted by NR. Treatment initiation estimates excluded 3463 patients with a recorded therapy starting prior to metastatic disease that continued beyond 14 days after the index date of metastatic disease. Overall survival estimates excluded 181 patients whose recorded death occurred before the index date of metastatic diagnosis. ICE=Index of Concentration at the Extremes.

## **Supplementary Table S4** Adjusted hazard ratios of treatment initiation and overall survival, stratified models by race and ethnicity

| **I. Adjusted hazard ratios of first-line treatment initiation [CI]** | | | | | |
| --- | --- | --- | --- | --- | --- |
| ICE Quintile | Latinx | Asian | Black | White | Other / Not documented |
| Q1: Least privileged | 0.743*** [0.609, 0.906] | 1.018 [0.714, 1.451] | 1.003 [0.835, 1.204] | 0.882*** [0.819, 0.951] | 0.966 [0.880, 1.061] |
| Q2 | 0.721*** [0.586, 0.888] | 1.076 [0.795, 1.457] | 1.057 [0.864, 1.293] | 0.881*** [0.832, 0.932] | 0.883*** [0.809, 0.964] |
| Q3 | 0.736*** [0.587, 0.923] | 0.955 [0.707, 1.289] | 1.040 [0.835, 1.296] | 0.887*** [0.842, 0.934] | 0.920* [0.843, 1.004] |
| Q4 | 0.857 [0.676, 1.087] | 0.758* [0.550, 1.043] | 0.978 [0.782, 1.223] | 0.911*** [0.867, 0.957] | 0.996 [0.913, 1.087] |
| Q5: Most privileged | Reference | Reference | Reference | Reference | Reference |
| **II. Adjusted hazard ratios of death [CI]** | | | | | |
| ICE Quintile | Latinx | Asian | Black | White | Other / Not documented |
| Q1: Least privileged | 1.053 [0.821, 1.352] | 0.869 [0.546, 1.381] | 1.132 [0.929, 1.380] | 1.149*** [1.057, 1.249] | 1.320*** [1.184, 1.471] |
| Q2 | 1.142 [0.885, 1.475] | 1.049 [0.705, 1.561] | 1.101 [0.884, 1.372] | 1.099*** [1.032, 1.172] | 1.144*** [1.034, 1.267] |
| Q3 | 0.977 [0.737, 1.296] | 1.031 [0.684, 1.552] | 1.089 [0.862, 1.377] | 1.121*** [1.058, 1.188] | 1.162*** [1.051, 1.285] |
| Q4 | 1.088 [0.809, 1.463] | 0.790 [0.516, 1.212] | 1.226* [0.964, 1.560] | 1.088*** [1.028, 1.150] | 1.018 [0.920, 1.127] |
| Q5: Most privileged | Reference | Reference | Reference | Reference | Reference |

Adjusted hazard ratios were estimated using the Cox model. 95% confidence intervals (CI) are reported in brackets. Adjusted hazard ratios were adjusted for age at metastatic diagnosis (continuous), calendar year of metastatic diagnosis (categorical), stage at initial diagnosis, molecular subtype, number of metastases, sites of metastasis and Eastern Cooperative Oncology Group performance status. Treatment initiation estimates excluded 3463 patients with a recorded therapy starting prior to metastatic disease that continued beyond 14 days after the index date of metastatic disease. Overall survival estimates excluded 181 patients whose recorded death occurred before the index date of metastatic diagnosis. SDOH=Social determinants of health. *** p<0.01, ** p<0.05, * p<0.10.

## **Supplementary Table S5. Adjusted hazard ratios of treatment initiation and overall survival using alternate constructions of ICE**

| **I. Adjusted hazard ratios of risk of first-line treatment initiation** [CI] | | | |
| --- | --- | --- | --- |
| Quintile | ICE: Low income Black Households versus high income White Households | ICE: Low income Households of Color versus high income White Households | ICE: Low income Latinx Households versus high income White Households |
| Q1: Least privileged | 0.905*** [0.863, 0.950] | 0.894*** [0.851, 0.939] | 0.872*** [0.830, 0.915] |
| Q2 | 0.890*** [0.852, 0.929] | 0.889*** [0.852, 0.928] | 0.907*** [0.869, 0.947] |
| Q3 | 0.897*** [0.861, 0.936] | 0.886*** [0.850, 0.924] | 0.904*** [0.867, 0.943] |
| Q4 | 0.928*** [0.891, 0.967] | 0.918*** [0.881, 0.956] | 0.928*** [0.891, 0.967] |
| Q5: Most privileged | Reference | Reference | Reference |
| **II. Adjusted hazard ratios of risk of death** [CI] | | | |
| Quintile | ICE: Low income Black Households versus high income White Households | ICE: Low income Households of Color versus high income White Households | ICE: Low income Latinx Households versus high income White Households |
| Q1: Least privileged | 1.170*** [1.107, 1.237] | 1.164*** [1.100, 1.231] | 1.143*** [1.081, 1.209] |
| Q2 | 1.124*** [1.070, 1.182] | 1.123*** [1.069, 1.180] | 1.122*** [1.068, 1.179] |
| Q3 | 1.126*** [1.073, 1.181] | 1.131*** [1.078, 1.186] | 1.145*** [1.092, 1.201] |
| Q4 | 1.076*** [1.027, 1.128] | 1.079*** [1.030, 1.131] | 1.066*** [1.018, 1.117] |
| Q5: Most privileged | Reference | Reference | Reference |

Adjusted hazard ratios were estimated using the Cox model. 95% confidence intervals (CI) are reported in brackets. Adjusted hazard ratios were adjusted for age at metastatic diagnosis (continuous), calendar year of metastatic diagnosis (categorical), stage at initial diagnosis, molecular subtype, number of metastases, sites of metastasis and Eastern Cooperative Oncology Group performance status. Treatment initiation estimates excluded 3463 patients with a recorded therapy starting prior to metastatic disease that continued beyond 14 days after the index date of metastatic disease. Overall survival estimates excluded 181 patients whose recorded death occurred before the index date of metastatic diagnosis. SDOH=Social determinants of health. *** p<0.01, ** p<0.05, * p<0.10.
